# The Influenza NS1 Protein: What Do We Know in Equine Influenza Virus Pathogenesis?

**DOI:** 10.3390/pathogens5030057

**Published:** 2016-08-31

**Authors:** Marta Barba, Janet M. Daly

**Affiliations:** 1 Department of Clinical Sciences, College of Veterinary Medicine, Auburn University, Auburn, AL 36849, USA; martabrvet@gmail.com; 2 School of Veterinary Medicine and Science, University of Nottingham, Sutton Bonington LE12 5RD, UK

**Keywords:** non-structural protein, equine influenza, pathogenicity, interspecies transmission

## Abstract

Equine influenza virus remains a serious health and potential economic problem throughout most parts of the world, despite intensive vaccination programs in some horse populations. The influenza non-structural protein 1 (NS1) has multiple functions involved in the regulation of several cellular and viral processes during influenza infection. We review the strategies that NS1 uses to facilitate virus replication and inhibit antiviral responses in the host, including sequestering of double-stranded RNA, direct modulation of protein kinase R activity and inhibition of transcription and translation of host antiviral response genes such as type I interferon. Details are provided regarding what it is known about NS1 in equine influenza, especially concerning C-terminal truncation. Further research is needed to determine the role of NS1 in equine influenza infection, which will help to understand the pathophysiology of complicated cases related to cytokine imbalance and secondary bacterial infection, and to investigate new therapeutic and vaccination strategies.

## 1. Introduction

Despite intensive vaccination programs in some horse populations, equine influenza virus (EIV) remains a serious health threat and potential economic problem throughout most parts of the world. Since 2000, several widespread EIV outbreaks have occurred, with the global transportation of horses responsible for introducing the virus into previously unexposed horse populations [1,2]. Equine influenza virus is an Orthomyxovirus of the genus influenza A and is an enveloped virus with a segmented, single-stranded, negative-sense ribonucleic acid (RNA) genome. The genome consists of eight segments that encode at least 10 proteins: the two surface glycoproteins hemagglutinin (HA) and neuraminidase (NA), from which the viral subtype is determined; three polymerase proteins; a nucleoprotein; two matrix proteins; a nuclear export protein; and non-structural protein 1 (NS1). Influenza viruses exist as genetically diverse populations, referred to as quasispecies, as a result of the high mutation rate caused by the error-prone replication of the viral RNA-dependent RNA polymerases [3]. The expansion of virus quasispecies provides the opportunity for viruses to evolve and escape from the host immune response and antiviral drugs [4]. 

Two different subtypes of EIV have been described, but the viruses currently circulating in horses are of the H3N8 subtype. The H3N8 subtype has diverged into two genetically and antigenically distinct lineages: Eurasian and American [5], and the American lineage subsequently diverged further into the Kentucky, South American and Florida sub-lineages [6]. The Florida sub-lineage has prevailed with the emergence of two clades: clade 1 viruses predominate in America, while clade 2 viruses predominate in Europe, but viruses of both clades have been associated with large EIV outbreaks on other continents [7]. 

Like all influenza viruses, after EIV enters the host cell, incoming viral ribonucleoproteins (vRNPs) are transported to the nucleus where two positive-sense RNAs are generated, messenger RNA (mRNA) and complementary RNA (cRNA) [8]. The cRNA is used as a template for producing progeny viral RNA. The newly synthesized vRNAs are exported to the cytoplasm where new viral particles are assembled and then exit the host cell by budding. The nuclear localization of influenza virus genome replication is uncommon for RNA viruses [9]. The influenza non-structural protein 1 (NS1) has multiple functions in the regulation of several cellular and viral processes during influenza infection and has been identified as an important virulence factor [10,11]. The NS1 protein facilitates viral replication by interfering with the host RNA processing machineries and their components by several mechanisms as a strategy for preferential viral RNA transcription [12,13]. However, this review focuses on the interactions of NS1 with proteins and RNA as a strategy for inhibiting cellular antiviral responses. 

**Figure 1:**
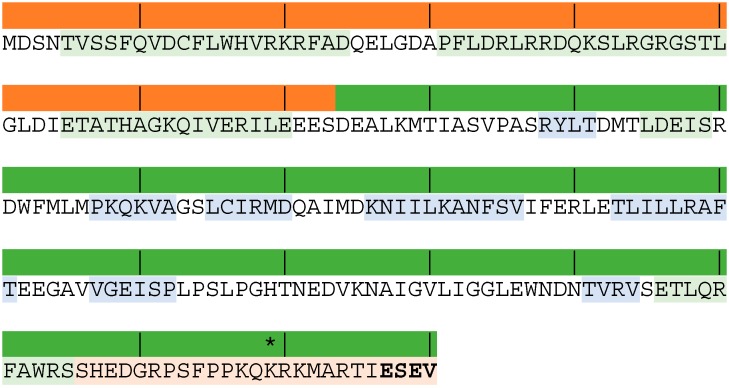
Predicted amino acid sequence of equine influenza strain A/equine/Uruguay/63 (H3N8) (GenBank accession number: CY032425). The N-terminal RNA-binding domain (residues 1–73) and C-terminal “effector” domain (residues 74–230) are indicated by the shaded bars above the amino acid sequence (red and green, respectively), with vertical lines marking every tenth amino acid. The green and blue text shading indicates the alpha and beta helices, respectively. The asterisk marks the position of the 11-amino-acid truncation seen in many recent equine influenza H3N8 isolates and the PDZ-binding domain (ESEV) is indicated in bold text.

The NS1 protein has two distinct functional domains (Figure 1): an N-terminal RNA-binding domain (residues 1–73) and a C-terminal “effector” domain (residues 74–230) [14,15]. The protein structure of each domain has been determined, but the final 20–25 amino acids appear to be intrinsically disordered. Interestingly, NS1 does not possess any intrinsic enzymatic activity. Its multifunctional nature is explained by the presence of epitopes that interact with many different host proteins and RNA, which is surprising for a protein that is only around 26 kDa [13]. Different epitopes bind different molecules for different functions: heterogeneous nuclear ribonucleoprotein E1B-AP5 (1–81 aa) [16]; double-stranded RNA (dsRNA, 19–38 aa) [17]; retinoic acid-inducible gene product I (RIG-I, 38–41 aa) [18]; nuclear localization signal 1 (NLS1, 34–38 aa) and 2 (NLS2, 216–221 aa) [19]; eukaryotic translation initiation factor 4GI (eIF4G, 81–113 aa) [12]; protein kinase R, (PKR, 123–127 aa) [20]; nuclear export signal (NES, 137–146 aa) [21]; cleavage and polyadenylation specific factor (CPSF, 103, 106, and 144–188 aa) [22]; poly-A binding protein II (PABPII, 223–230 aa) [23]; and (PDZ, 227–230 aa) [24]. Several other protein-binding regions are still undetermined. NS1 exists as a homodimer and both of the functional domains contribute to the stability and dimerization of the NS1 protein, which is essential for its multiple functions [25,26]. Different dimerization conformations of the effector domain have been discovered and it has been hypothesized that they can occur in a strain- or ligand-specific manner, which can contribute to the multifunctional nature of the NS1 protein [13].

## 2. Influenza NS1 Protein Inhibition of Host Antiviral Response

Elevated inflammatory cytokine responses, such as tumor necrosis factor (TNF)-α or interleukin-6 (IL-6), have been related to the severe pathology associated with highly pathogenic influenza viruses in humans [27,28]. Type I interferons, IFN-α and β, are produced by many different cell types during the innate immune response to influenza virus infection and are involved in the recruitment of inflammatory cells to the site of infection, which creates an antiviral state in the host cell to limit virus spread and growth. IFN production is induced by recognition of pathogen-associated molecular pattern (PAMP) receptors, which then activate the main IFN regulatory transcription factors IRF-3 and NF-κB, which in turn transactivate IFN-β gene expression [29]. Influenza viruses have developed mechanisms to evade these host defense mechanisms and one of the major functions of the viral NS1 protein is to antagonize the host type I IFN response. It has been proposed that NS1 limits the production of IFN-β both by pre-transcriptional and post-transcriptional mechanisms in a strain-dependent manner [13].

Pre-transcriptionally, NS1 prevents dsRNA- and virus-mediated activation of Interferon Regulatory Factor 3 (IRF-3), NFκB and c-Jun/ATF-2 transcription factors [29,30,31]. NS1 targets the retinoic acid-inducible gene product I (RIG-I) signaling pathway involved in the transcriptional activation of the IFN-β gene [18]. NS1 has also been described to inhibit two IFN-inducible gene products, protein kinase R (PKR) and 2′,5′-oligoadenylate synthetase (OAS) [17,32]. Both proteins use dsRNA as a cofactor in their activation, and together they can impair virus replication by creating an antiviral state within cells. The sequestering of dsRNA by NS1 has been reported to prevent their activation [17,32]. In addition, NS1 was shown to bind PKR in a RNA-binding–independent manner by binding with the cellular PACT protein and blocking PACT/RIG-I–mediated activation of type I IFN [20]. Further activities of the NS1 protein include the activation of phosphoinoside-3-kinase (PI3K) signaling, which downregulates apoptosis [33]. The four C-terminal amino acids of full-length NS1 proteins have a canonical X[S/T]XV (where X = any amino acid) class I PDZ-binding motif (Figure 1) [24]. PDZ domains are protein-protein recognition modules within a multitude of proteins that organize diverse cell-signaling assemblies and it is thought that the PDZ-binding motif expressed may have an impact on the virulence by disrupting certain cellular pathways [24]. 

The NS1 protein of some influenza strains is also thought to enhance pathogenicity by enabling them to replicate despite high levels of cytokine production [13]. One study showed that replication of an H5N1 virus strain was resistant to the antiviral effects of pro-inflammatory cytokines; this resistance required glutamic acid at amino acid position 92 of NS1 [34].

## 3. What Do We Know about Equine Influenza NS1?

As the NS1 gene is more conserved than the highly variable HA, several studies have focused on phylogenetic analysis of NS1 to provide insight into the epidemiological dynamics of influenza viruses. Large-scale phylogenetic analyses have suggested that the equine influenza A viruses have resulted from the direct introduction of viruses from avian hosts [35,36]. Two distinct alleles of the NS1 gene (allele A and B) circulate in aquatic birds, the reservoir host species for all known influenza A virus subtypes. The majority of influenza A viruses that have become established in mammalian hosts have an NS1 that belongs to allele A [37]. An exception is A/equine/Jilin/89 (H3N8), which was independently transmitted to horses from an avian source in an outbreak in China [38].

**Figure 2:**
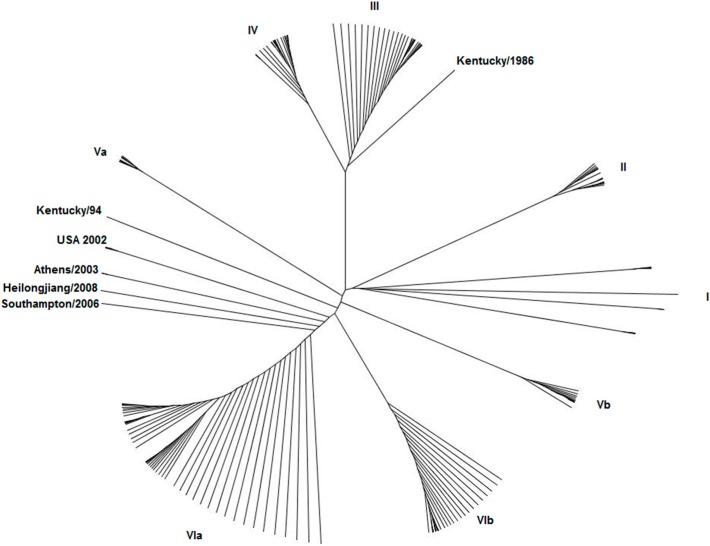
Phylogenetic tree of equine influenza non-structural NS1 proteins. Amino acid sequences of 77 equine influenza virus NS1 proteins were downloaded from the Influenza Research Database and the phylogeny inferred using Phylogeny.fr [40]. Clusters of strains labeled with Roman numerals are described in the text. Outlier strains are individually labeled.

It appears that the HA and NS genes of equine influenza evolve in a parallel fashion, leading to classification of NS1 into the same five groups that are used for the HA classification: the pre-divergent lineage, the Eurasian and American-Kentucky sub-lineages, and the Florida sub-lineage clades 1 and 2 [39]. In a phylogenetic analysis of all equine influenza H3N8 subtype equine-predicted NS1 amino acid sequences currently available in the Influenza Research Database, further sub-clusters are revealed (Figure 2). The clusters labeled I–III contain the pre-divergent strains. Cluster I includes the prototype equine influenza A H3N8 subtype virus Miami/63 and the other three earliest equine H3N8 strains isolated in South America from 1963 to 1969. Cluster II includes pre-divergence strains isolated between 1975 and 1980 and cluster III includes strains isolated from 1981 to 1989. Clusters IV and V arose as a result of divergent evolution on the Eurasian and American continents [5] and represent the Eurasian and American-Kentucky groups, respectively. Clusters VIa and VIb represent Florida clades 2 and 1, respectively. Sequencing of the NS1 gene in addition to the HA has revealed the occurrence of reassortment, for example the UK isolate A/equine/Lincolnshire/1/06 was a member of clade 2 of the Florida sub-lineage according to the HA sequence but a member of the Eurasian lineage according to the NS sequence [7].

**Figure 3:**
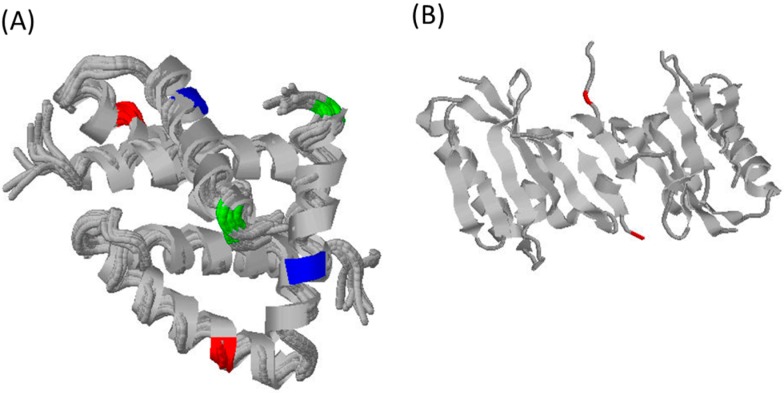
Cartoon representations of NS1 structure. Amino acid changes typically found in Florida sub-lineage viruses are indicated in: (**A**) the RNA-binding domain in red (amino acid, aa44), blue (aa59) and green (aa71); and (**B**) the effector domain in red (aa86). Images were created using RasMol for Windows v2.7.5.2 and PDB files 1NS1 (**A**) and 3DR (**B**).

Florida sub-lineage viruses are distinguished by amino acid changes at positions 44, 59 and 71 in the RNA-binding domain (Figure 3A), 86 in the effector domain (Figure 3B) and 216, which lies at the juncture of the effector domain and the disordered C-terminal region. Florida sub-lineage viruses are also characterized by an 11-amino-acid truncation due to the introduction of a stop codon. 

Naturally occurring NS1 proteins with C-terminal truncations of 11 to 30 amino acids due to non-sense mutations are common in mammalian influenza viruses [13]. In addition to the 11-amino-acid truncation seen in the majority of recent equine influenza strains, three isolates in cluster I (two isolated in Japan in 1971 and one from Algiers in 1972) have a 13-amino-acid truncation. In general, the effect of the NS C-terminal truncation is the attenuation of the virus due to inefficiency in blocking host gene expression, leading to higher levels of the antiviral responses such as type I IFN and other pro-inflammatory cytokines [11,13,41,42]. Several studies have investigated the effects of NS1 truncation by constructing truncated NS1 recombinant viruses with the same backbone to minimize variability due to the effect of differences between changes in other influenza genes among strains. In one study, the NS1 truncation of a laboratory-adapted human influenza strain (A/WSN/33 [H1N1]) led to the increased host gene expression of inflammatory cytokines such as IL-6 and TNF-α, in human macrophages and in mice [43]. Similarly, another study demonstrated that NS1 truncation decreased the ability of swine influenza viruses to prevent type I IFN synthesis in pig cells [11]. Furthermore, these mutant viruses were attenuated in vivo in pigs. Interestingly, the 2009 flu pandemic in people was caused by a novel swine-origin H1N1 influenza virus that possessed an 11 aa C-terminal truncation in its NS1 protein, which was associated with a higher induction of pro-inflammatory cytokine response than human seasonal H1N1 viruses [42]. It was demonstrated that the 11 aa truncation was responsible for inefficient blocking of host gene expression, nucleolar localization, and PABII-binding capacity because the NSL-2– and PABII-binding domain are present at the C-terminal region of NS1 (Figure 1). The NS1 truncation did not significantly alter virus replication in vitro or in vivo, but surprisingly enhanced virus virulence in mice. 

The original equine H3N8 isolates (Miami/63, Uruguay/63 and Sao Paulo/6/1963) have an ESEV PDZ-binding motif at amino acids 227–230. This may reflect the recent avian origin of these strains as ESEV is the consensus avian PDZ-binding motif [44]. All but three of the strains in cluster II also have an ESEV motif whereas the only strain with an ESEV motif not found in cluster I or II is A/equine/Switzerland/P112/2007, which is in cluster IV and is an example of so-called ‘frozen evolution’ (see below). The majority of strains isolated post-divergence with full-length NS1 proteins have an EPEV at the terminal four amino acids. A different motif is seen in five strains; four strains isolated in the USA between 1994 and 1997 end in the sequence EPEI and one from the USA in 2003 ends in KPKI. The change in the PDZ-binding motif from ESEV to EPEV may be a result of adaptation of the virus to a mammalian host as the motifs found in the PDZ-binding domain contribute to viral pathogenicity in a species-dependent manner [45]. A recent study demonstrated that the ESEV motif found in the majority of currently circulating HPAI H5N1 viruses specifically associated with the PDZ proteins Scribble, Dlg1, MAGI-1, MAGI-2, and MAGI-3 whereas the EPEV motif did not [44]. The downstream effect of binding Scribble and Dlg1 was the disruption of cellular tight junctions in a mammalian cell line; thus, the ESEV motif may contribute to the greater pathogenicity of an avian virus in a mammalian host. 

Several equine influenza isolates have anachronous NS1 sequences: A/equine/La Plata/88 is in cluster I as is A/equine/Essaouira/3/2004, but this strain has an EPEV motif; A/equine/Heilongjiang/1/2010 is in cluster II with an ESEV motif; A/equine/Cheshire/1/06 is clustered with the older American lineage strains, which were not detected in the UK since 1998; and A/equine/Switzerland/P122/07 is clustered with older Eurasian lineage viruses. Whether these examples of frozen evolution are a result of a reduced evolutionary rate in some circulating equine influenza viruses or due to some or all of the gene segments of older viruses being reintroduced into circulation (e.g., as a result of inadvertent release of the virus from a laboratory or reassortment with a vaccine strain) is not clear. Bryant et al. (2009) suggested the latter could be the case for A/equine/Cheshire/1/06 and A/equine/Switzerland/P122/07 as they have similar HA and NS1 gene sequences to the references strains A/equine/Newmarket/1/93 and A/equine/Sussex/89, respectively [7]. 

A recent Florida clade 1 H3N8 isolate from horses in Korea was found to have an internal deletion of 23 bases (from 327 to 349) of the NS1, leading to a frame shift which, in addition to the C-terminal truncation, resulted in an NS1 protein only 117 amino acids long. This virus was attenuated in cell culture compared to A/equine/Miami/63 (full-length NS1), which was associated with increased expression of antiviral and pro-inflammatory genes [46].

## 4. Potential Role of NS1 in Equine Pathology and Inter-Species Transmission

Recently, it was demonstrated that the H3N8 equine influenza virus has jumped the species barrier to infect dogs on more than one occasion and spread among canine populations in the US [47,48,49,50]. A representative equine influenza strain with a truncated NS1 (A/equine/Newmarket/5/03) was demonstrated to be significantly more effective at inducing type I interferon activity in infected ponies than earlier strains (A/equine/Sussex/1989 or A/equine/Newmarket/2/93) [51]. The A/equine/Newmarket/5/03 strain was also associated with a longer duration of coughing in horses. These factors may have influenced the ability of the recently circulating equine influenza strains to cross the species barrier and may be linked to the truncated NS1 protein, although pathogenicity is a highly multifactorial process with most influenza proteins playing a role.

It is unknown if equine influenza viruses could overcome species barriers such as sialic acid receptor specificity and potentially infect people [42]. The most recent human influenza pandemic in 2009 was caused by an H1N1 strain originating from the reassortment of viruses from different species and included gene segments of swine, human, and avian origin. It is hypothesized that polymorphisms in NS1, including truncation and the presence of PDZ sequences, could have an important role in virulence observed during pandemic outbreaks [13,23,42]. Although the earliest H3N8 canine influenza viruses to be identified in Florida in 2003 and 2004 had a truncated NS1, all canine H3N8 viruses characterized since 2005 have possessed the full-length NS protein, conserving an intact PDZ domain [52,53]. This may be an example of adaptive mutation in the NS1 gene in order to switch hosts.

Understanding the role of NS1 in the pathophysiology of influenza infection could help to manage complicated cases. For example, secondary bacterial infection is the major cause of mortality after primary influenza infection [54,55]. Differences in NS1 have been related to the increased production of cytokines in humans and in horses. The cytokine dysregulation associated with previous influenza infection is linked with secondary bacterial infection in humans, mice, and pigs [56]. Further research is needed to investigate the role of equine influenza infection and bacterial pleuropneumonia, especially regarding the innate immune response and virulence factors, such as NS1.

## 5. Potential to Apply Knowledge about NS1 for Prevention and Control of Influenza

One interesting application derived from the creation of recombinant NS1-attenuated viruses is the development of live attenuated virus vaccines. These have the advantage of stimulating both humoral and cell-mediated immunity. In humans and swine, NS1-attenuated viruses generated by a reverse genetics approach have been investigated for vaccine production, with the additional advantage that the relevant surface antigens (HA and NA) can readily be replaced in the master strain in order to update the vaccine strains [11,57]. The effect of further C-terminal truncation of the NS1 gene of the prototype Florida clade II strain A/equine/Kentucky/5/02 was studied using a plasmid-based reverse genetics system to generate recombinant equine influenza viruses [58]. The truncated viruses were attenuated in their ability to block the antiviral response, with higher amounts of IFNβ being produced in infected cells compared to wild-type recombinant viruses. Accordingly, NS1 C-terminal truncation led to replication attenuation of the viruses in cell and egg culture, as well as in mice [58]. Surprisingly, the recombinant viruses with the shortest NS1 protein were the least attenuated [58]. Seronegative horses were vaccinated with three of the NS1 truncated viruses. Horses vaccinated with one (NS1-126) did not develop fever, had significantly fewer clinical signs and had significantly reduced quantities of virus shedding for a shorter duration post-challenge compared to unvaccinated controls [59]. However, reliance on the mutation of a single gene to generate an attenuated virus means that there is a risk of reversion to virulence or the acquisition of compensatory mutations that enable the virus to resist the antiviral effects of pro-inflammatory cytokines.

As the NS1 protein shows less strain variation than HA and NA, it may be a better therapeutic target for antiviral therapy [60]. Various approaches are being used to identify small molecules that might target the interactions between NS1 and host proteins or dsRNA in order to restore the function of innate immunity and inhibit virus replication. Further research is needed to evaluate the potential use of these drugs, but they could be a good option for use in horses, considering the public health risk of using antiviral drugs such as the NA inhibitor oseltamivir, where rapid development of resistance is observed, in horses.

## 6. Concluding Remarks

It has been demonstrated that NS1 strain-specific differences are important in regulating host-cell responses triggered by infection, which can lead to differences in pathology and virulence in different species including humans, pigs, and equids. Variation in the NS1 protein occurs by various mechanisms including truncations and deletions and reassortment. The quasispecies nature of influenza A viruses, including EIV, may facilitate their adaptation to new hosts as variants with point mutations leading to changes that are non-lethal to the virus but affect how the virus interacts with the host’s innate immune response which can be rapidly selected from the quasispecies cloud. Understanding the role of polymorphisms in equine NS1 could lead to better prediction of the likely impact of emerging strains in the horse and other mammalian species and improve prevention and control measures.

## Abbreviations

The following abbreviations are used in this manuscript:

CPSF	Cleavage and Polyadenylation Specific Factor
eIF4G	eukaryotic translation initiation factor 4GI
EIV	equine influenza virus
HA	hemagglutinin
IFN	interferon
IL	interleukin
NA	neuraminidase
NES	nuclear export signal
NLS	nuclear localization signal
NS1	non-structural protein 1
OAS	2′,5′-oligoadenylate synthetase
PABPII	poly-A binding protein II.
PAMPs	pathogen-associated molecular patterns
PI3K	phosphoinoside-3-kinase
PKR	protein kinase R
RIG-I	retinoic acid-inducible gene product I
TNF	tumor necrosis factor
TRIM	tripartite motif
